# A Case of Sebaceous Adenitis and Concurrent Meibomian Gland Dysfunction in a Dog

**DOI:** 10.3390/vetsci7020037

**Published:** 2020-04-02

**Authors:** Roberta Sartori, Claudio Peruccio

**Affiliations:** 1 Servizi Dermatologici Veterinari, 20131 Milan, Italy; 2 Veterinary Ophthalmology Referrals, Centro Veterinario Torinese, 10153 Turin, Italy; claudio.peruccio@yahoo.it

**Keywords:** meibomian gland dysfunction, meibomitis, sebaceous adenitis

## Abstract

Sebaceous adenitis and concurrent meibomian gland dysfunction (MGD) were diagnosed in a two-year-old mongrel dog presenting with hypotrichosis, exfoliative dermatitis and blepharitis. Diagnosis of sebaceous adenitis was based on history, clinical signs, the histological demonstration of multifocal lymphohistiocytic and neutrophilic inflammation targeting the sebaceous glands and sebaceous glands loss. MGD was diagnosed by non-contact infrared meibography followed by tear film lipid layer interferometric evaluation. Ciclosporin and sebolytic shampoos controlled the dermatological condition, while doxycycline, warm compresses, palpebral massages and tobramycin/dexamethasone ointment controlled the blepharitis. This case report should stimulate clinicians to investigate MGD in dogs suffering from sebaceous adenitis, because the meibomian and sebaceous glands share similar anatomy and physiology.

## 1. Introduction

The ophthalmologic evaluation of a patient with sebaceous adenitis and concurrent blepharitis is here reported. This examination is always recommendable, since meibomian gland dysfunction (MGD) can be a comorbidity due to the anatomic, physiologic and embryologic similarities between the sebaceous glands and meibomian glands (MGs) [1]. To our knowledge, this is the first report of sebaceous adenitis and concurrent MGD in a dog.

## 2. Case Description

**Figure 1:**
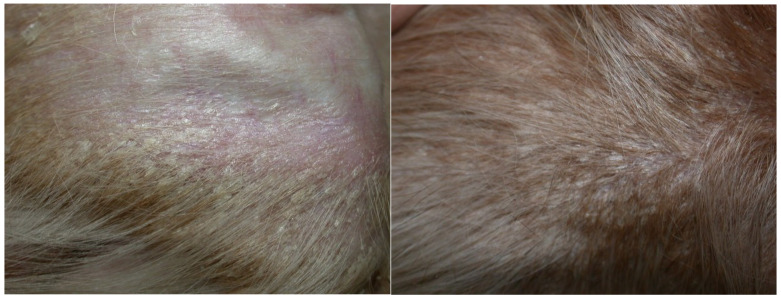
Hypotrichosis, follicular casts and exfoliative dermatitis on the concave surface of the pinna (**left**) and on the flank (**right**). Scales are fine, whitish, adherent and non-adherent, with an average diameter of 0.3–0.5 cm.

**Figure 2:**
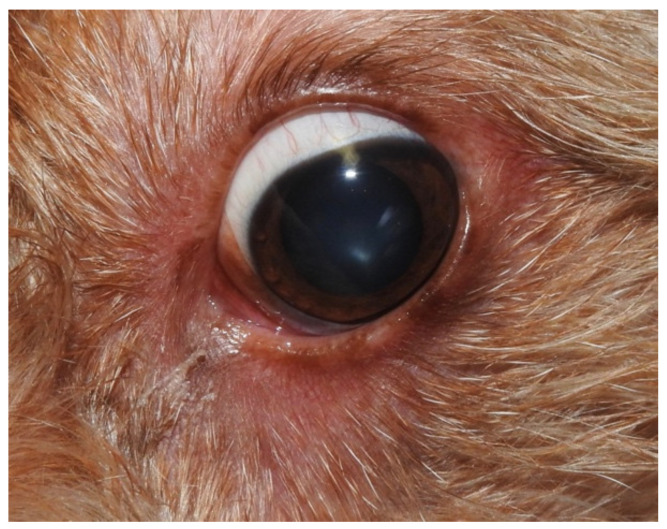
Blepharitis and conjunctivitis with a wet eye.

A two-year-old neutered female mongrel dog was presented for the evaluation of non-pruritic diffuse hypotrichosis, erythema and exfoliative dermatitis of five months’ duration. Clinical data reported by the referring veterinarian included negative serology for leishmaniosis (immunofluorescence antibody test) and unremarkable haematological and biochemical findings. The dog was therefore treated twice monthly with 4% chlorexidine shampoos (Clorexyderm shampoo; I.C.F., Cremona, Italy) and fatty acid supplementation (Omega Pet Perle; NBF Lanes, Milano, Italy), without any improvement, for three months. A dermatological consultation was then requested. On general examination, the dog was normal. Dermatological examination revealed moderate erythema on the pinnae, dorsum, ventral neck, thorax and abdomen. Generalised hypotrichosis was present and it was more apparent on the concave surfaces of pinnae, ventral neck and flanks. Follicular casts on the apex of both pinnae were present. Multifocal areas of exfoliative dermatitis were localised on the dorsum, flanks and pinnae (Figure 1). Mild conjunctivitis with epiphora and periocular erythema were also affecting both eyes (Figure 2). The hair coat was of poor quality. The problems of the dog were defined as erythematous and exfoliative generalised dermatitis and hypotrichosis.

**Figure 3:**
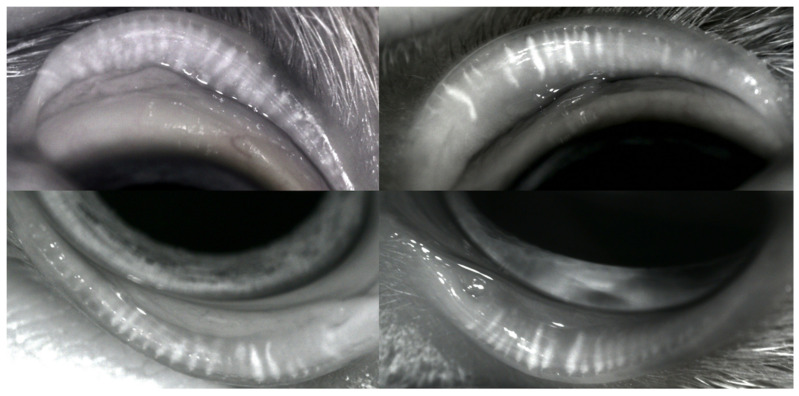
Non-contact infrared meibography. **Right eye**: meibomian glands (MGs) appear thinner and shorter, some are filled with more opaque dense secretions and some show an interrupted outline. **Left eye**: MG changes are even more evident, with substantial atrophy and dropout.

The differential diagnoses included sebaceous adenitis, epitheliotropic cutaneous lymphoma, leishmaniosis and keratinisation defects. As far as hypotrichosis was concerned, endocrine dysfunctions were also considered as differentials. Further blood exams (fT4, tT4 and TSH) and the urine cortisol/creatinine ratio were requested; no thyroid dysfunction was found. The cortisol/creatinine ratio was normal. Therefore, the dog was anaesthetised, and four 6 mm skin biopsy punch samples were taken from the hypotrichotic and exfoliative regions of the flanks and pinnae. The skin biopsy samples were formalin fixed and routinely processed. Histological examination revealed mild to moderate, diffuse, regular epidermal hyperplasia and severe lamellar to basketwave orthokeratotic hyperkeratosis with follicular plugging. Multifocal, moderate, lymphohistiocytic and neutrophilic sebaceous adenitis and sebaceous gland loss were apparent in the dermis. A diagnosis of sebaceous adenitis was made based on the histopathological examination. The dog was therefore treated with topical therapy consisting of a twice-weekly sebolytic shampoo containing zinc gluconate and salicylic acid (Sebolytic shampoo; Virbac, Milano, Italy) and the application of a skin moisturiser (Johnson’s Baby Oil; Johnson & Johnson, Roma, Italy). In addition to the topical therapy, ciclosporin (Atoplus; Elanco, Sesto Fiorentino, Italy) at 5 mg/kg once daily was administered. At the recheck, after two months, the skin condition was improving, but bilateral blepharoconjunctivitis with mucopurulent discharge was present, so an ophthalmic consultation was requested. A complete eye examination was performed. The dog’s eyes were red, with mucopurulent discharge and continuous blinking. Epiphora was apparent. The Schirmer Tear Test (STT) result was >30 mm/min in the right eye and 28 mm/min in the left. The MGs, examined by slit-lamp, were thinner, and shorter, with a significant reduction in number and ductal occlusion. Ocular surface stainings by Fluorescein and Rose Bengal were mildly positive in both eyes, the ocular media were transparent and the ocular fundus of both eyes was normal. A diagnosis of MGD and mucopurulent conjunctivitis was made, and an accurate check of the ocular surface (OS), tear film (TF) and MGs was suggested. TF examination was carried out by non-contact infrared meibography and interferometry, both performed by a hand held ocular surface analyser, OSA-Vet^®^ (SBM Sistemi, Torino, Italy). MGs were altered with local dilatation, opaque content, shortening, atrophy and dropout; ductal openings were occluded as a consequence of scarring and terminal ductules were plugged with an inspissated secretion (Figure 3). The TF lipid layer was thin, about 20–30 nm thick in both eyes, the Tear Meniscus Height (TMH) was low (0.29 mm right eye, 0.27 mm left eye), and the OS topography examined with the Placido disc was normal. Tear osmolarity was 360 mOsm/L in the right eye and 357 mOsm/L in the left. The treatment of ophthalmic disorders included twice-daily warm compresses and palpebral massages, as well as tobramycin/dexamethasone (TobraDex; Novartis Farma, Milano, Italy) ointment and systemic doxycycline (Vibravet; Zoetis, Roma, Italy) at 10 mg/kg once daily for three weeks. Sebolytic shampoos and moisturisers were continued twice weekly.

**Figure 4:**
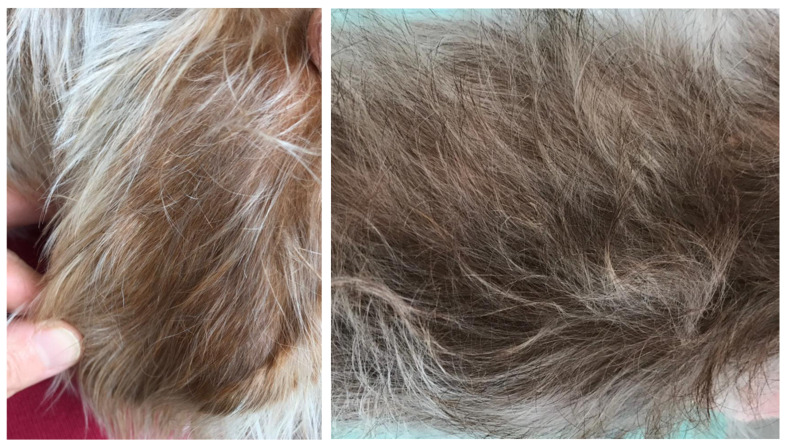
The absence of lesions on the pinna (**left**) and on the flank (**right**).

**Figure 5:**
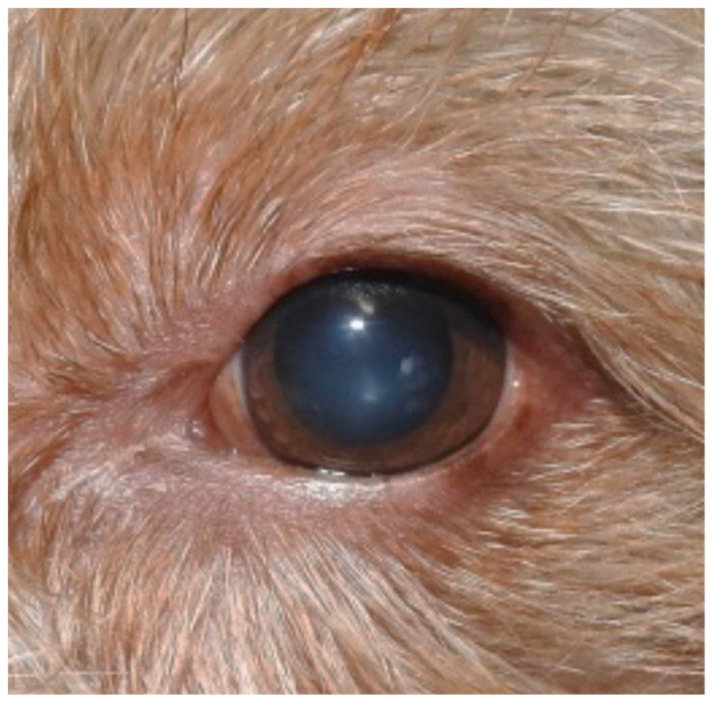
Remission of the clinical signs of blepharitis.

At the recheck, after three weeks, both dermatologic (Figure 4) and ophthalmologic (Figure 5) clinical signs improved. Tear osmolarity was 333 mOsm/L in the right eye and 292 mOsm/L in the left. MGs, examined by meibography, were irreversibly altered, with ductal occlusion due to MGD. Despite that, there was a moderate change in the estimate of lipid layer thickness (30–40 nm), and TMH was increased (0.45 mm right eye and 0.57 mm left eye).

At the time of writing, the dog was treated by the systemic administration of ciclosporin every three days and once-to-twice-monthly shampooing, and her condition was in remission.

## 3. Discussion

Sebaceous adenitis is an uncommon alopecic and scaling dermatosis described in dogs, and more rarely, in other mammals [2]. Lymphocytic granulomatous or pyogranulomatous inflammation targets and destroys the sebaceous glands, leading to prominent scaling and follicular plugging, as presented in this case. These are due to lack of sebum production. As far as the pathogenesis of the disease is concerned, both immune-mediated attacks on the sebaceous glands and keratinization disorders have been hypothesised [2]. The case presented here showed all of the clinical characteristics previously described in the literature. In addition, ocular changes related to MGD were apparent. This finding has never been reported in association with sebaceous adenitis before, but it may be a frequent and underestimated comorbidity. In fact, the MGs are large sebaceous glands that are likely to be affected by inflammation and destruction in a patient suffering from sebaceous adenitis. These glands are located in the eyelids and are not associated with hairs, although their embryologic ectodermal development shows considerable similarities to that of the hair follicle [1]. The central meibomian duct may be compared to the hair follicles of the eyelashes in embryology and also shows distinct similarities in structure and epithelial differentiation, including keratinisation [1]. The production of lipids is followed by the occurrence of keratohyalin granules in the luminal epithelial cells. Lipids produced by the MGs are the main component of the superficial lipid layer (LL) of the tear film protecting it against the evaporation of the aqueous phase and are also believed to stabilise it by lowering the surface tension. Hence, meibomian lipids are essential to the maintenance of OS health and integrity [1]. The glands are under neural and hormonal control and secrete their oil into shallow reservoirs on the lid margins. Functional disorders of the MG alter the LL, decrease its stability, and increase aqueous phase evaporation from the ocular surface, which, eventually, results in a rise of tear osmolarity [3]. The ophthalmic clinical signs of a red, wet eye are a consequence of the evaporative dry eye (EDE) condition secondary to MGD. When MG secretion decreases and the meibum composition is altered, the LL is thin, tear evaporation increases—causing an increase in osmolarity—and, as a compensatory process, the lacrimal glands increase their tear production. Hence, EDE secondary to MGD is often characterised by a high STT result but a low TMH due to the lack of meibomian lipids spread over the eyelid margin, with a loss of the barrier containment of tears and a consequent wet eye. MGD is a chronic, diffused abnormality of the MGs that may result in TF alteration, eye irritation, inflammation and OS disease [3]. MGD is a common underlying pathology in dogs with OS disorders [4]. These signs were apparent, in combination with dermatological alterations, in the case here described. Several factors contribute to MGD pathogenesis, such as an increase in meibum viscosity, inflammatory mediators, bacterial lipid-degrading enzymes, hormonal changes, and topical or systemic medications [4,5,6]. In the case here presented, histopathology of MGs was not performed because the excision of eyelids is an invasive procedure, and no inflammatory infiltrate similar to that found in sebaceous adenitis was demonstrated, but it was the most likely differential. Non-contact infrared meibography showed abnormal MG morphology and ductal obstruction; the LL, examined by interferometry, turned out to be thin—a potential cause of increased TF evaporation and dry eyes. As far as sebaceous adenitis was concerned, both topical and systemic therapies were chosen, with an emphasis on topical treatment. In fact, topical treatment in addition to systemic ciclosporin administration is reported to reduce scaling more effectively than ciclosporin alone. Moreover, there is evidence of a synergistic benefit on both scaling and alopecia when the two treatment options are combined. Inflammation of the sebaceous glands is also best reduced by a combination of both ciclosporin and topical therapy [7].

It is not known whether systemic ciclosporin administration could have affected the MGD outcome. Only topical ciclosporin, in literature, is reported to have a beneficial effect on MGD [8]. The use of systemic ciclosporin in this disease has been discussed and it does not seem to benefit human patients [8]. Unfortunately, literature about dogs is lacking.

## 4. Conclusions

The complete ophthalmologic evaluation of a patient with sebaceous adenitis and concurrent blepharitis is always recommendable, since MGD can be a comorbidity due to the anatomic and physiologic similarities between the sebaceous glands and MGs. Future prospective studies are needed to support our findings.
